# Autosomal recessive cerebellar ataxia caused by mutations in the *PEX2 *gene

**DOI:** 10.1186/1750-1172-6-8

**Published:** 2011-03-10

**Authors:** Caroline Sevin, Sacha Ferdinandusse, Hans R Waterham, Ronald J Wanders, Patrick Aubourg

**Affiliations:** 1 Pediatric Neurology and Endocrinology, Hôpital St Vincent de Paul, Paris, France; 2 University of Amsterdam, Academic Medical Centre, Departments of Clinical Chemistry and Pediatrics, Laboratory Genetic Metabolic Diseases, Amsterdam, The Netherlands

## Abstract

**Objective:**

To expand the spectrum of genetic causes of autosomal recessive cerebellar ataxia (ARCA).

**Case report:**

Two brothers are described who developed progressive cerebellar ataxia at 3 1/2 and 18 years, respectively. After ruling out known common genetic causes of ARCA, analysis of blood peroxisomal markers strongly suggested a peroxisomal biogenesis disorder. Sequencing of candidate *PEX *genes revealed a homozygous c.865_866insA mutation in the *PEX2 *gene leading to a frameshift 17 codons upstream of the stop codon. *PEX *gene mutations usually result in a severe neurological phenotype (Zellweger spectrum disorders).

**Conclusions:**

Genetic screening of PEX2 and other PEX genes involved in peroxisomal biogenesis is warranted in children and adults with ARCA.

## Background

Main causes of autosomal recessive cerebellar ataxia (ARCA) include Friedreich ataxia, ataxia telangiectasia and oculomotor apraxia type 1 and 2 [1,2]. Cerebellar ataxia may sometimes be the leading neurologic symptom in a limited number of autosomal recessive metabolic diseases [1,2] but has not been reported as such in inherited peroxisomal diseases due to isolated enzyme deficiency or peroxisomal biogenesis defect [3,4]. Patients with adrenomyeloneuropathy, the adult form of X-adrenoleukodystrophy, or adult Refsum disease (resulting from mutations in PHYH gene coding for the peroxisomal phytanoyl-CoA hydroxylase enzyme, or in PEX7 gene coding for the peroxin 7 receptor protein) often develop cerebellar ataxia but have always associated neurological symptoms (peripheral neuropathy, retinitis pigmentosa, spastic paraplegia) [5,6]. Patients with peroxisomal biogenesis defects (Zellweger spectrum disorders) may also display cerebellar dysfunction among many other neurological symptoms when they have prolonged survival [3,4]. Peroxisomal biogenesis disorders (PBDs) are characterized by the loss of multiple peroxisomal metabolic functions caused by mutation in 13 PEX genes involved in the import of peroxisomal matrix proteins [3,4]. Except for rhizomelic chondrodysplasia punctata due to mutations in the *PEX7 *gene, all patients have multiple metabolic impairments involving: (1) oxidation of very long chain fatty acids (VLCFA), branched-chain fatty acids (phytanic and pristanic acids, bile acid intermediates) and L-pipecolic acid; (2) and plasmalogen biosynthesis. We report two brothers who developed isolated progressive cerebellar ataxia at 3 1/2 and 18 years. After ruling out common genetic causes of ARCA, screening of peroxisomal metabolites revealed in the youngest brother at 9 years a moderate increase in the plasma levels of phytanic and pristanic acids suggesting PBD. This patient was afterwards found to have a homozygous mutation in the *PEX2 *gene. His older brother carrying the same *PEX2 *gene mutation developed cerebellar ataxia at 18 years.

## Case presentation

The younger brother (P1) was evaluated for progressive gait disturbance at the age of 9 years. He was the fourth child of non-consanguineous healthy parents. His milestones were normal but mild dysarthria was noticed at 2 1/2 years. Gait disturbance appeared at 3 1/2 years. At 7 years, he walked with a wide-based gait but was able to run, rise from the floor and climb stairs. On neurological examination, the child had moderate truncal ataxia, dysarthria but no tremor and pyramidal signs in the lower limbs. Symptoms worsened at the age of 9 years: he was no longer able to run, could walk only 100 meters without support and had difficulties with writing. On the Wechsler Intelligence Scale for Children (WISC-III), his full, verbal and performance IQ were 75, 84 and 79. He had truncal ataxia, moderate cerebellar tremor, mild scanning dysarthria, nystagmus, slow saccades without oculo-motor apraxia and hyporeflexia. His total ataxia score evaluated by the International Cooperative Ataxia Rating Scale (ICARS) was 17/100. He had no other neurological symptoms and his physical examination was normal. Cerebellar ataxia exacerbated slowly up to the age of 14 years but he was still able to walk without support. The ICARS score was 33/100. No other neurological signs were present and cognitive function remained normal.

**Figure 1:**
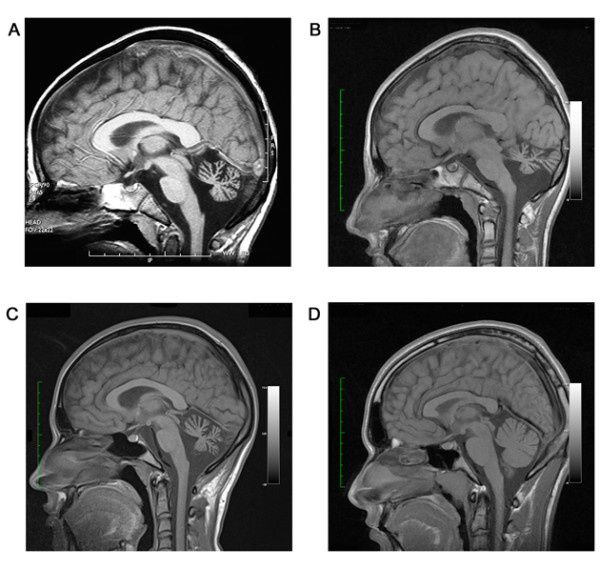
**Brain T-1 weighted magnetic resonance imaging showing marked cerebellar atrophy in P1 at the age of 9 years (panel A) and 14 years (panel B), and in P2 at the age of 18 years (panel C)**. Panel D shows a normal brain MRI in a 16 years-old control.

At the age of 9 years, a brain MRI showed atrophy of the vermis and lateral hemispheres of the cerebellum that worsened at the age of 14 years, but without signs of demyelination or neuronal migration defects in forebrain and cerebellum (Figure 1). Fundoscopic examination, electroretinogram, EEG, electromyography, peripheral nerve conduction, visual, brainstem auditory and somatosensory evoked potentials, and audiogram were normal at 9 and 14 years. Other normal tests included ECG, cardiac ultrasound, liver enzymes, ammonia, lactate, vitamin E, lipid electrophoresis, alpha-foeto protein, plasma and urinary amino acids and urinary organic acids. Molecular studies of frataxin and aprataxin genes ruled out Friedrich ataxia and cerebellar ataxia with oculomotor apraxia 1 (AOA1).

His older brother (P2) had normal neurologic examination at 14 years, but at the age of 18 years he started to develop cerebellar signs with impaired gait, dysmetria, ataxia of the trunk, dysarthria, hyporeflexia and slow saccades with oculomotor apraxia. The ICARS score was 17/100. He had no other neurological symptoms. On the WISC-IV, his full, verbal and performance IQ were 80, 74 and 75 (normal range). Brain MRI showed isolated cerebellar atrophy at 18 years (Figure 1). Electroretinogram, peripheral nerve conduction, visual, brainstem auditory, and somatosensory evoked potentials were normal. These two patients had a 7-year-old brother and two sisters, aged of 22 and 25 years, who had a normal neurologic examination.

After ruling out Friedrich ataxia, AOA1 and common metabolic causes of cerebellar ataxia, peroxisomal parameters were analysed in plasma of patient P1 and his brother and sisters. Plasma VLCFAs were nearly normal in patients P1 and P2, but a moderate increase in the levels of phytanic and pristanic acids was found, prompting us to suspect a peroxisomal disorder (Table 1). Further studies showed a moderate increase of bile acid intermediates (DHCA and THCA) in plasma and normal levels of pipecolic acid in plasma and CSF (Table 1).

**Table 1:** Analysis of plasma VLCFAs, branched-chain fatty acids and bile acids intermediates.

	Controls (5% - 95% range)	Patient 1	Patient 2
▪ **VLCFAs**			
- C22:0	40-119	34	36
- C24:0	33-84	33	38
- C26:0	0.45-1.32	0.76	0.95
- C24:0/C22:0	0.57-0.92	0.98	1.07
- C26:0/C22:0	0-0.02	0.02	0.03
▪ **Branched-chain fatty acids**			
- Phytanic acid	0-9	23.5	10.3
- Pristanic acid	0-4	27.9	16.7
- Pristanic/Phytanic ratio	0.05-0.4	1.19	1.62
▪ **Bile acids intermediates**			
- DHCA	0-0.02	0.4	0.1
- THCA	0-0.08	0.1	0.2

All values are given in μM.

The findings of a moderate elevation of pristanic and phytanic acids with normal VLCFAs in plasma from P1 and P2 suggested initially that they might suffer from α-methylacyl-CoA racemase (AMACR) deficiency [7]. This was ruled out by the measurement of R- and S- isomers of DHCA and THCA in plasma and AMACR activity in fibroblasts from P1. The presence of DHCA and THCA traces also excluded the diagnosis of adult Refsum disease. Further metabolic studies performed in fibroblasts [7,8] from P1 showed normal plasmalogen biosynthesis, DHAP-AT activity, oxidation of VLCFAs, and pristanic acid (Table 2). Oxidation of phytanic acid was only slightly abnormal. Taken together, those analyses pointed towards a mild peroxisomal defect, but did not clearly indicate at which level. Since few patients with *PEX2*, *PEX10 *and *PEX12 *gene mutations display a relatively mild PBD phenotype, a complete genomic sequencing of these genes was performed. No mutations were identified in the *PEX10 *and *PEX12 *gene, but an apparent homozygous c.865_866insA mutation was found in the *PEX2 *gene in patients P1 and P2. The mother was heterozygous for the c.865_866insA mutation. Paternal genotype could not be determined because the proband's father had lost contact with the family. No mutation was found in P1 and P2 asymptomatic siblings, who had normal peroxisomal parameters in plasma and no neurologic symptoms.

**Table 2:** Biochemical abnormalities in fibroblasts from patient 1 and 5 other patients with PEX2 mutations.

	Controls (5% - 95% range)	Patient 1	Other PEX2 deficient patients				
			1	2	3	4	5
▪ **Peroxisomal beta-oxidation**^**a**^							
- C26:0	1214-1508	1296	186	1172	98	78	215
- Pristanic acid	675-1121	1114	2	495	41	1	23
▪ **Phytanic acid alpha-oxidation**^**a**^	39-97	28	5	ND	2	1	15
▪ **Plasmalogen de novo synthesis**							
- %pPE in PE	72.8-81.4	79.6	58.5	0.8	17.4	10.4	35.4
- %pPC in PC	3.3-5.5	5.8	1.1	0.5	2.8	1.3	4.2
▪ **DHAPAT-activity**^**b**^	5.8-12.3	8.3	0.7	7.8	0.6	1.3	`0.8
▪ **Catalase immunofluorescence**	+	+	-	+/-	-	-	-
▪ **Immunoblot analysis**							
- Acyl-CoA oxidase (70/50/20 kDa)	+/+/+	+/+/+	+/-/-	+/+/+	+/-/-	+/-/-	+/-/-
- Peroxisomal thiolase (44/41 kDa)	+/+	+/+	+/-	+/-	+/-	+/-	+/-

Patients 1-4 are described in ref 12. Patient 5 is a newly identified PEX2 patient with severe clinical phenotype. ^a^pmol/(h.mg protein); ^b^nmol/(2 h.mg protein).

**List of abbreviations**: DHAP-AT, dihydroxyacetonephosphate-acyltransferase; DHCA, dihydroxycholestanoic acid; PC, total phosphatidylcholine; pPC, plasmalogen phosphatidylcholine; PE, total phosphatidylethanolamine; pPE, plasmalogen phosphatidylethanolamine; THCA, trihydroxycholestanoic acid; VLCFA, very-long chain fatty acids.

## Discussion

The *PEX2*, *PEX10 *and *PEX12 *genes encode peroxins that are integral peroxisomal membrane proteins with a cytosolic carboxy-terminal RING finger domain that act as ubiquitin ligases required for the ubiquitination of the PTS1-receptor (PEX5) in the peroxisomal membrane [9].

We do not have any reliable background data on the frequency of the c.865_866insA mutation in the proband's ethnic background, and this mutation has not been reported in the database that we have consulted (http://www.hgmd.cf.ac.uk; http://www.dbpex.org; http://www.ncbi.nlm.nih.gov/snp). However, because the c.865_866insA mutation introduces a frameshift 17 codons upstream of the stop codon, resulting in a PEX2 protein with an altered C-terminus (p.Ser289LysfsX36), it is very unlikely that it could be a non-pathogenic variation occurring in the normal population. Additionally, homozygosity for this mutation co-segregated undeniably with the disease in the family. It should be noted, however, that homozygosity for this mutation could not be confirmed due to the unavailability of paternal DNA. This leaves the possibility that the paternal mutant allele harbors a different mutation, for example a (partial) deletion of the PEX2 gene, which will not be recognized when in trans with the c.855_866insA allele. In that case the c.855_866insA allele will also appear as homozygous.

The fact that peroxisomes in the patient's fibroblasts were normally present and contained catalase suggests that the mutated PEX2 is localized correctly in the peroxisomal membrane and still is partly active. How this moderate impairment in peroxisome biogenesis impairs selectively neuronal cells in cerebellum is unknown. It is possible that environmental factors and modifier genes contribute to the different age of onset of clinical symptoms in the two affected brothers.

Cerebellar defects (cerebellar hypoplasia, altered folial pattern, abnormal Purkinje cell dendritic arborization) are observed in PEX 2 deficient mice [10]. Tissue-selective elimination of peroxisomes in the mouse's brain or liver suggests that the folial abnormalities may be due mainly to the lack of hepatic peroxisomes, whereas the Purkinje cells defects may imply both brain and liver peroxisomal abnormal metabolism [11]. The patients that we describe seem to have progressive cerebellar atrophy rather than malformative cerebellar hypoplasia.

The clinical phenotype of these two patients strongly differs from PBD patients with *PEX2 *gene mutations who may display cerebellar symptoms in addition to other severe neurological signs in the context of Zellweger spectrum disorders [12,13].

While this work was in progress, PBD patients with mild phenotypes including cerebellar ataxia were shown to have mutations in *PEX10 *gene or *PEX16 *gene [14,15]. However, these patients displayed additional neurological symptoms including axonal motor neuropathy and decreased vibration sense in *PEX10*- mutated patients and spastic paraparesia, leukodystrophy peripheral neuropathy and cataracts in patients with *PEX16 *mutation. In contrast, our patients have a milder phenotype restricted to isolated cerebellar ataxia.

## Conclusion

These clinical observations broaden the spectrum of phenotypes associated with PEX gene mutations but, most importantly, indicate that a search for PEX gene defect must be considered as potential cause of ARCA. Systematic screening for such PEX gene defect can easily and reliably be performed by the simple measurement of peroxisomal metabolites in plasma and will probably increase the frequency of PBD patients with mild phenotypes such as ARCA.

## Consent

Written consent was obtained from the patient P2 and parents of patient P1 for publication of this case report.

## Competing interests

The authors declare that they have no competing interests.

## Authors' contributions

CS and PA were involved in the clinical evaluation and follow-up of the patient, the data analysis and interpretation, and drafted the manuscript. SF, HRW and RJW carried out the biochemical and molecular genetic studies and the interpretation of the results. SF was involved in the write-up of the manuscript. All authors read and approved the final manuscript.

## References

[B1] Fogel BL Perlman S Clinical features and molecular genetics of autosomal recessive cerebellar ataxias Lancet Neurol 2007 6 245 257 10.1016/S1474-4422(07)70054-6 17303531

[B2] Manto M Marmolino D Cerebellar ataxias Curr Opin Neurol 2009 22 419 429 10.1097/WCO.0b013e32832b9897 19421057

[B3] Steinberg SJ Dodt G Raymond GV Braverman NE Moser AB Moser HW Peroxisome biogenesis disorders Biochim Biophys Acta 2006 1763 1733 1748 10.1016/j.bbamcr.2006.09.010 17055079

[B4] Wanders RJ Metabolic and molecular basis of peroxisomal disorders: a review Am J Med Genet A 2004 126 355 375 10.1002/ajmg.a.20661 15098234

[B5] Moser HW Mahmood A Raymond GV X-linked adrenoleukodystrophy Nat Clin Pract Neurol 2007 3 140 151 10.1038/ncpneuro0421 17342190

[B6] Wanders RJ Jansen GA Skjeldal OH Refsum disease, peroxisomes and phytanic acid oxidation: a review J Neuropathol Exp Neurol 2001 60 1021 1031 11706932 10.1093/jnen/60.11.1021

[B7] Ferdinandusse S Denis S Clayton PT Graham A Rees JE Allen JT McLean BN Brown AY Vreken P Waterham HR Wanders RJ Mutations in the gene encoding peroxisomal alpha-methylacyl-CoA racemase cause adult-onset sensory motor neuropathy Nat Genet 2000 24 188 191 10.1038/72861 10655068

[B8] Ebberink MS Mooyer PA Koster J Dekker CJ Eyskens FJ Dionisi-Vici C Clayton PT Barth PG Wanders RJ Waterham HR Genotype-phenotype correlation in PEX5-deficient peroxisome biogenesis defective cell lines Hum Mutat 2009 30 93 98 10.1002/humu.20833 18712838

[B9] Girzalsky W Saffian D Erdmann R Peroxisomal protein translocation Biochim Biophys Acta 2010 1803 724 731 10.1016/j.bbamcr.2010.01.002 20079383

[B10] Faust PL Su HM Moser A Moser HW The peroxisome deficient PEX2 Zellweger mouse: pathologic and biochemical correlates of lipid dysfunction J Mol Neurosci 2001 16 289 97 10.1385/JMN:16:2-3:289 11478384

[B11] Krysko O Hulshagen L Janssen A Schütz G Klein R De Bruycker M Espeel M Gressens P Baes M Neocortical and cerebellar developmental abnormalities in conditions of selective elimination of peroxisomes from brain or from liver J Neurosci Res 2007 85 58 72 10.1002/jnr.21097 17075904

[B12] Gootjes J Elpeleg O Eyskens F Mandel H Mitanchez D Shimozawa N Suzuki Y Waterham HR Wanders RJ Novel mutations in the PEX2 gene of four unrelated patients with a peroxisome biogenesis disorder Pediatr Res 2004 55 431 436 10.1203/01.PDR.0000106862.83469.8D 14630978

[B13] Shimozawa N Zhang Z Imamura A Suzuki Y Fujiki Y Tsukamoto T Osumi T Aubourg P Wanders RJ Kondo N Molecular mechanism of detectable catalase-containing particles, peroxisomes, in fibroblasts from a PEX2-defective patient Biochem Biophys Res Commun 2000 5 31 5 10.1006/bbrc.1999.2082 10652207

[B14] Regal L Ebberink MS Goemans N Wanders RJ De Meirleir L Jaeken J Schrooten M Van Coster R Waterham HR Mutations in PEX10 are a cause of autosomal recessive ataxia Ann Neurol 2010 68 259 263 20695019 10.1002/ana.22035

[B15] Ebberink MS Csanyi B Chong WK Denis S Sharp P Mooijer PA Dekker CJ Spooner C Ngu LH De Sousa C Wanders RJ Fietz MJ Clayton PT Waterham HR Ferdinandusse S Identification of an unusual variant peroxisome biogenesis disorder caused by mutations in the PEX16 gene J Med Genet 2010 47 608 15 10.1136/jmg.2009.074302 20647552

